# Supplementary material for: Using synchronized brain rhythms to bias memory-guided decisions
Source: eLife. 2024 Jul 22;12:RP92033. doi: 10.7554/eLife.92033 (PMC11262798; doi:10.7554/eLife.92033)
Supplement: Supplementary file 1. — (a) Statistics from the delayed alternation brain-machine interfacing experiment from Figure 2. (b) Statistics from Figure 3H showing change in medial prefrontal cortex (mPFC)-hippocampal theta coherence difference scores (high coherence – low coherence trials) as rats navigated toward and away from the choice point infrared beam. (c) Statistics from Figure 5 power analysis. (d) Statistics from Figure 5 coherence analysis. (e) Multivariate granger prediction results (Figure 5). [file elife-92033-supp1.docx]

## Supplementary File 1a

Statistics from the delayed alternation brain machine interfacing experiment from **Fig. 2**.

| *Test* | *X* | *Y* | *T-stat* | *df* | *Conf. Interval* | *p-val* | *# corrections* |
| --- | --- | --- | --- | --- | --- | --- | --- |
| *ttest* | High | Y.High | 2.8 | 7 | [2.62, 28.3] | 0.02 | 0 |
| *ttest* | Low | Y.Low | -0.3 | 7 | [-16.5, 13.2] | 0.80 | 0 |
| *ttest* | High | Rand | 6.1 | 7 | [10.4, 23.4] | 0.002 | 3 |
| *ttest* | Y.High | Rand | 0.32 | 7 | [-9.2, 12.1] | 0.76 | 0 |
| *ttest* | Low | Rand | 0.8 | 7 | [-7.6, 15.6] | 0.44 | 0 |
| *ttest* | Y.Low | Rand | 1.3 | 7 | [-4.7, 16.0] | 0.24 | 0 |

*N = 8 rats. Bonferroni’s method was used to correct p-values for multiple comparisons if a significant effect was observed. The experiment was designed to compare coherence trials to yoked control trials and as such, these comparisons were planned.*

## Supplementary File 1b

Statistics from Fig. 3H showing change in mPFC-hippocampal theta coherence difference scores (high coherence – low coherence trials) as rats navigated towards and away from the choice-point infrared beam.

| ***Time from choice (seconds)*** | ***t-stat*** | ***p-value*** | ***FDR p-value*** |
| --- | --- | --- | --- |
| *-1.86* | 3.71 | 0.01 | 0.04 |
| *-1.57* | 2.83 | 0.03 | 0.06 |
| *-1.29* | 1.92 | 0.10 | 0.12 |
| *-1.00* | 0.27 | 0.79 | 0.79 |
| *-0.71* | 0.58 | 0.58 | 0.66 |
| *-0.43* | 3.46 | 0.01 | 0.04 |
| *-0.14* | 2.45 | 0.04 | 0.07 |
| *0.14* | 2.41 | 0.05 | 0.07 |
| *0.43* | 3.23 | 0.01 | 0.04 |

*N = 8 rats. FDR correction achieved with Benjamini Hochbergs method.*

## Supplementary File 1c

Statistics from **Fig. 5** power analysis

| Test | X | Y | T-stat | df | Conf. Interval | p-val | # corrections |
| --- | --- | --- | --- | --- | --- | --- | --- |
| ttest | PF | 0 | 14.7 | 21 | [0.025, 0.033] | <0.001 | 3 |
| ttest | VMT | 0 | 5.02 | 21 | [0.009, 0.02] | <0.001 | 3 |
| ttest | HC | 0 | 9.6 | 21 | [0.008, 0.01] | <0.001 | 3 |

*N = 22 sessions distributed across 3 rats. p-values were corrected via Bonferroni’s method when significance was reported. T-tests were performed against a null of 0 or against a paired dataset. PF = mPFC, VMT = Ventral midline thalamus, HC = Hippocampus.*

## Supplementary File 1d

Statistics from **Fig. 5** coherence analysis

| Test | X | Y | T-stat | df | Conf. Interval | p-val | # corrections |
| --- | --- | --- | --- | --- | --- | --- | --- |
| ttest | PF-VMT | 0 | 9.2 | 21 | [0.28, 0.44] | <0.001 | 3 |
| ttest | VMT-HC | 0 | 5.2 | 21 | [0.06, 0.14] | <0.001 | 3 |
| ttest | PF-VMT | VMT-HC | 5.6 | 21 | [0.17, 0.37] | <0.001 | 3 |

*N = 22 sessions distributed across 3 rats. p-values were corrected via Bonferroni’s method when significance was reported. T-tests were performed against a null of 0 or against a paired dataset. PF = mPFC, VMT = Ventral midline thalamus, HC = Hippocampus.*

## Supplementary File 1e

Multivariate granger prediction results (**Fig. 5**).

| Test | X | Y | Tstat | df | Conf. Interval | p-val | # corrections |
| --- | --- | --- | --- | --- | --- | --- | --- |
| ttest | HC2VMT | 0 | 2.06 | 21 | [-0.00, 0.12] | 0.05 | 0 |
| ttest | VMT2HC | 0 | 0.32 | 21 | [-0.05, 0.06] | 0.76 | 0 |
| ttest | HC2VMT | VMT2HC | 1.26 | 21 | [-0.03, 0.13] | 0.22 | 0 |
| ttest | PF2VMT | 0 | 7.46 | 21 | [0.13, 0.22] | 0.00** | 3 |
| ttest | VMT2PF | 0 | 2.87 | 21 | [0.02, 0.11] | 0.03* | 3 |
| ttest | PF2VMT | VMT2PF | -3.66 | 21 | [-0.17, -0.05] | 0.00** | 3 |
| ttest | PF2HC | 0 | 7.56 | 21 | [0.21, 0.36] | 0.00** | 3 |
| ttest | HC2PF | 0 | 7.58 | 21 | [0.25, 0.43] | 0.00** | 3 |
| ttest | PF2HC | HC2PF | 1.01 | 21 | [-0.17, 0.06] | 0.33 | 0 |

*N = 22 sessions distributed across 3 rats. p-values were corrected via Bonferroni’s method when significance was reported. *p<0.05. **p<0.01. T-tests were performed against a null of 0 or against a paired dataset. PF = mPFC, VMT = Ventral midline thalamus, HC = Hippocampus.*
